# Individual response in patient’s effort and driving pressure to variations in assistance during pressure support ventilation

**DOI:** 10.1186/s13613-023-01231-9

**Published:** 2023-12-20

**Authors:** Mattia Docci, Emanuele Rezoagli, Maddalena Teggia-Droghi, Andrea Coppadoro, Matteo Pozzi, Alice Grassi, Isabella Bianchi, Giuseppe Foti, Giacomo Bellani

**Affiliations:** 1 School of Medicine and Surgery, University of Milano-Bicocca, Monza, Italy; 2 Department of Emergency and Intensive Care, Fondazione IRCCS San Gerardo Dei Tintori, Monza, Italy; 3 Department of Anesthesia and Pain Medicine, Toronto General Hospital, Toronto, ON Canada; 4 Department of Anesthesia and Intensive Care, ASST Papa Giovanni XXIII, Bergamo, Italy; 5 Centre for Medical Sciences-CISMed, University of Trento, Trento, Italy; 6 Department of Anesthesia and Intensive Care, Santa Chiara Hospital, APSS Trento Largo Medaglie d’Oro Trento, Trento, Italy

**Keywords:** Acute respiratory failure, Artificial ventilation, Pressure support ventilation, Monitoring, Breathing effort, Pressure muscle index, Driving pressure, Respiratory system compliance

## Abstract

**Background:**

During Pressure Support Ventilation (PSV) an inspiratory hold allows to measure plateau pressure (Pplat), driving pressure (∆P), respiratory system compliance (Crs) and pressure-muscle-index (PMI), an index of inspiratory effort. This study aims [1] to assess systematically how patient’s effort (estimated with PMI), ∆P and tidal volume (Vt) change in response to variations in PSV and [2] to confirm the robustness of Crs measurement during PSV.

**Methods:**

18 patients recovering from acute respiratory failure and ventilated by PSV were cross-randomized to four steps of assistance above (+ 3 and + 6 cmH_2_O) and below (-3 and -6 cmH_2_O) clinically set PS. Inspiratory and expiratory holds were performed to measure Pplat, PMI, ∆P, Vt, Crs, P0.1 and occluded inspiratory airway pressure (Pocc). Electromyography of respiratory muscles was monitored noninvasively from body surface (sEMG).

**Results:**

As PSV was decreased, Pplat (from 20.5 ± 3.3 cmH_2_O to 16.7 ± 2.9, *P* < 0.001) and ∆P (from 12.5 ± 2.3 to 8.6 ± 2.3 cmH_2_O, *P* < 0.001) decreased much less than peak airway pressure did (from 21.7 ± 3.8 to 9.7 ± 3.8 cmH2O, *P* < 0.001), given the progressive increase of patient’s effort (PMI from -1.2 ± 2.3 to 6.4 ± 3.2 cmH_2_O) in line with sEMG of the diaphragm (*r* = 0.614; *P* < 0.001). As ∆P increased linearly with Vt, Crs did not change through steps (*P* = 0.119).

**Conclusion:**

Patients react to a decrease in PSV by increasing inspiratory effort—as estimated by PMI—keeping Vt and ∆P on a desired value, therefore, limiting the clinician’s ability to modulate them. PMI appears a valuable index to assess the point of ventilatory overassistance when patients lose control over Vt like in a pressure-control mode. The measurement of Crs in PSV is constant—likely suggesting reliability—independently from the level of assistance and patient’s effort.

**Supplementary Information:**

The online version contains supplementary material available at 10.1186/s13613-023-01231-9.

## Background

Spontaneous breathing during acute respiratory failure carries both advantages and drawbacks [1]. In patients with milder forms of acute respiratory distress syndrome (ARDS), it is associated with less need of sedation [2], prevention of diaphragm myofiber damage and atrophy [3] and increased venous return to right atrium improving right ventricle preload and stroke volume [4]. Conversely, some data in animal models and in patients support the patient self-inflicted lung injury hypothesis suggesting that spontaneous breathing may cause lung injury due to excessive respiratory effort, leading to large tidal volumes (Vt) and injurious transpulmonary pressures [5–10]. Hence, monitoring the activity of patient’s respiratory muscles during spontaneous breathing is crucial.

The standard method to measure the inspiratory muscle pressure generated by the patient is based on esophageal pressure (Pes) [11, 12]. Electrical activity of the crural diaphragm signal represents an alternative tool to measure respiratory drive and, indirectly effort [13, 14]. Although such techniques are accurate, their application in daily clinical practice is still limited by devices availability, technical issues, the need for a solid background physiological knowledge and the lack of a clear evidence on the impact on patients’ outcome [15–17].

To overcome this gap, some methods have been developed to measure inspiratory effort, non-invasively, at the bedside. First, most ventilators allow to perform a brief inspiratory hold during pressure support ventilation (PSV). This may unveil the inspiratory effort generated by the patient in addition to the pressure support delivered by the ventilator [18]. The difference between end-inspiratory occlusion plateau pressure (Pplat) and peak airway pressure (Ppeak) before occlusion, also known as pressure-muscle-index (PMI), has a moderate-to-strong correlation with Pes-derived pressure time product [19]. The sum of set PS and PMI equals the driving pressure (∆P) imposed on the respiratory system, which is associated with outcome in patients with ARDS on PSV [20, 21].

Second, diaphragmatic surface electromyography (sEMG) is a novel promising non-invasive tool to monitor electrical activity of the diaphragm at the costal margin and accessory muscles through a few external electrodes [22, 23].

This study has two main aims. First, to assess the changes in patient’s inspiratory effort (estimated with PMI), ∆P and the corresponding Vt after variations of ventilatory assistance (PS level). These measurements are corroborated by measurements of sEMG of the diaphragm and intercostal muscles together with P0.1 [24] and Pocc a more recently described established index of effort [25, 26]. Second, we aimed to further confirm the validity of the measurement of respiratory system compliance (Crs) during PSV, under the hypothesis that ∆P depends solely on Vt irrespectively from the level of assistance applied or patient’s inspiratory effort.

## Materials and methods

We performed a single-center prospective crossover randomized physiological study in patients admitted to the General Intensive Care Unit (ICU) of San Gerardo Hospital, Monza. The institutional ethics committee (Azienda Socio Sanitaria Territoriale Monza, Italy) approved the study on 11/05/2020 (number 3269) according to the ethical principles of the Declaration of Helsinki and to the good clinical practice of the Ministero della Sanità (15/07/1997). Informed written consent was obtained from each patient, except during the SARS-CoV2 pandemic emergency, according to Italian Law (“Decreto del Ministero della Sanità”, 15 July 1997). If patient gave verbal consent but was unable to sign the form, signature of an independent witness was obtained.

### Patient selection

In the timeframe from July 2020 to May 2022, we enrolled in this study a convenience sample of 18 patients with the following inclusion criteria: age greater or equal to 18 years old, clinical diagnosis of Acute Hypoxic Respiratory Failure, ventilated in PSV mode via endotracheal tube or tracheal cannula. Exclusion criteria were pregnancy and breastfeeding, neurological and neuromuscular diseases, Acute Exacerbation of Chronic Obstructive Pulmonary Disease or acute asthmatic attack, clinical contraindications to variations in PSV level, psychomotor agitation or need of high-dose sedation and contraindication or impossibility to sEMG positioning (such as confirmed phrenic nerve lesion or open abdomen and open chest treatment).

### Study design

Demographic data (age, sex, Body Mass Index), pre-existing comorbidities and primary diagnosis responsible for the need for mechanical ventilation were extracted from the Electronic Medical Record (Innovian, Draeger, Germany). Patients baseline characteristics were assessed collecting bedside clinical data (i.e., arterial pressure, heart rate, peripheral oxygen saturation and Richmond Agitation-Sedation Scale) [27] together with the need of sedation or vasopressors, blood samples for arterial blood gas analysis (PaO_2_/FiO_2_) and biochemistry (including platelets count, creatinine, bilirubin) to obtain Sequential Organ Failure Assessment (SOFA) Score [28].

For the study protocol (Additional file 1: Figure A1), we cross-randomized each patient to four PS levels, two above (i.e. + 3 and + 6 cmH2O) and two below (i.e., −3 and −6 cmH2O) the clinically set PS level, keeping positive end-expiratory pressure (PEEP) constant. Steps were randomized using a simple sequence written within envelopes opened blindly by the attending physician in charge of the studied patient. During each 10-min step, including the one at clinically set PS, after clinical stability and a regular respiratory pattern were reached we collected the following respiratory parameters from the ventilator (Evita XL, Drägerwerk AG & Co., Lubeck, Germany) as the mean value over 15 breaths: mean airway pressure, Vt, respiratory rate (RR), minute ventilation (MV). These data were stored and analyzed off-line using LabChart 7 Pro (ADInstruments, Sidney, Australia). Vt coefficient of variation was then computed as the ratio between SD and mean Vt over the 15 breaths. At the end of each step, we performed one expiratory hold and one inspiratory hold (or more if needed until plateau pressure reliability criteria [29] were satisfied) to calculate respiratory mechanics and breathing effort parameters such as Ppeak, Pplat, PMI, ∆P, Crs, P0.1 and Pocc. During each step, electrical activity of both the diaphragm at the costal margin (EADi,surf) and the intercostal inspiratory muscles (intercost,surf) was continuously monitored via a dedicated surface electromyography device (sEMG Recorder, Drägerwerk AG & Co., Lubeck, Germany). Ventilator and electromyography waveforms during an inspiratory hold in a representative patient are reported in Additional file 1: Figure A2 in the online Additional Content.

### Surface electromyography

Details on electrodes positioning (Additional file 1: Figure A3), signal acquisition, processing and analysis are discussed in the online Additional Content.

### Statistical analysis

Normality of data distribution was assessed using Shapiro–Wilk test. Data were reported as mean ± SD or median and IQR as appropriate. Categorical data were reported as count (proportion). Differences between continuous variables were tested across PS levels using repeated measurements one-way ANOVA or Friedman test, as appropriate. Patients were further divided by lower and higher respiratory system compliance subgroups using the median value of Crs at clinical PS. We applied this stratification since we reasoned that the impact of a different level of assistance would be different based on patient’s Crs, especially towards higher levels of assistance. Differences between continuous variables in the two Crs subgroups were tested across PS levels using mixed ANOVA and the level of significance of the group by time interaction was reported. Post-hoc comparisons between groups at each PS step were explored using the two-stage linear step-up procedure of Benjamini, Krieger and Yekutieli. Correlations between EADI,surf versus p0.1 and PMI and between PMI versus P0.1 and Pocc were tested by Spearman or Pearson correlation coefficient as appropriate. 95% confidence interval was estimated using a thousand repetitions of bootstrapping resampling. Statistical significance was considered when P-value < 0.05. Statistical analysis was performed using Prism GraphPad 8.3.0. software and Stata/MP 17.0 for Mac (StataCorp LLC, StataCorp, College Station, TX 77845, USA).

## Results

### Study population

We enrolled 18 patients undergoing PSV invasively. There were no missing data. Demographics and baseline characteristics are presented in Additional file 1: Table A1 (60% were females, age was 60 ± 14 years old with a BMI of 28 ± 8 kg/m^2^). Patients were no longer in the most severe phase of illness with a median of 6 days from intubation (IQR [4;12]), but still under light sedation (Richmond Agitation-Sedation Scale equal to -1 [−2;0]). SOFA score was 6 [3;8], PaO2/FiO_2_ ratio was 264 ± 84 mmHg, with no major hemodynamic or acid–base disorders. Table 1 reports respiratory variables collected at the different PS levels tested (clinical PS, ± 3 cmH_2_O and ± 6 cmH_2_O). During the study, the PS level ranged from 1.8 ± 2.1 to 13.8 ± 2.1 cmH_2_O, while extrinsic PEEP was left unchanged (7.8 ± 2.6 cmH_2_O).

**Table 1:** Respiratory variables at the different pressure supports tested

	−6 cmH_2_O	−3 cmH_2_O	Clinical PS	+ 3 cmH_2_O	+ 6 cmH_2_O	P-value
PS, cmH_2_O	1.8 ± 2.1	4.8 ± 2.1	7.8 ± 2.1	10.8 ± 2.1	13.8 ± 2.1	< 0.001
PEEP, cmH_2_O	7.8 ± 2.6	7.8 ± 2.6	7.8 ± 2.6	7.8 ± 2.6	7.8 ± 2.6	N/A
Vt, L	0.42 ± 0.11	0.45 ± 0.10	0.46 ± 0.10	0.53 ± 0.13	0.59 ± 0.17	< 0.001
Vt, mL/Kg	6.9 ± 1.5	7.4 ± 1.5	7.8 ± 1.4	8.9 ± 2.0	10.0 ± 2.6	< 0.001
Vt coefficient of variation	0.15 ± 0.08	0.14 ± 0.09	0.12 ± 0.05	0.09 ± 0.05	0.07 ± 0.04	< 0.001
MV, L/min	8.6 ± 1.8	8.5 ± 1.5	8.6 ± 1.4	8.3 ± 1.4	8.9 ± 1.6	0.663
RR, breaths/min	22 ± 4	20 ± 4	19 ± 5	17 ± 4	15 ± 5	< 0.001
RSBI, breaths/min/L	52 ± 19	44 ± 17	43 ± 23	33 ± 13	27 ± 16	< 0.001
Ppeak, cmH_2_O	9.7 ± 3.8	12.7 ± 3.8	15.7 ± 3.8	18.7 ± 3.8	21.7 ± 3.8	< 0.001
MAP, cmH_2_O	8.2 ± 2.8	8.9 ± 2.5	10.3 ± 3.1	10.5 ± 2.5	11.3 ± 2.7	< 0.001
Pplat, cmH_2_O	16.7 ± 2.9	17.4 ± 3.5	18.1 ± 2.6	19.1 ± 2.8	20.5 ± 3.3	< 0.001
∆P, cmH_2_O	8.6 ± 2.3	9.5 ± 2.4	10.1 ± 1.8	11.2 ± 1.9	12.5 ± 2.3	< 0.001
Crs, mL/cmH_2_O	51 ± 13	49 ± 13	47 ± 13	48 ± 12	48 ± 13	0.119
PMI, cmH_2_O	6.4 ± 3.2	4.7 ± 3.2	2.3 ± 2.6	0.4 ± 2.2	−1.2 ± 2.3	< 0.001
Pocc, cmH_2_O	−14.8 ± 6.4	−10.7 ± 6.4	−7.9 ± 4.5	−5.5 ± 4.3	−4.8 ± 3.5	< 0.001
P0.1, cmH_2_O	−2.5 ± 1.2	−2.0 ± 1.3	−1.4 ± 0.8	−1.0 ± 0.7	−0.8 ± 0.8	< 0.001
EADi,surf, %clin	191 ± 78	134 ± 61	100 ± 0	77 ± 31	70 ± 27	< 0.001
Intercost,surf, %clin	150 ± 51	114 ± 29	100 ± 0	70 ± 26	74 ± 38	< 0.001

Data are presented as mean ± SD. P-values shown correspond to one-way ANOVA for repeated measurements significance level. Surface electromyography of diaphragm (EADi,surf) and intercostal muscles (Intercost,surf) is expressed as percentage of the signal at clinical PS (%clin). *PS* pressure support, *PEEP* positive end expiratory pressure, *Vt* tidal volume, *MV* minute ventilation, *RR* respiratory rate, *RSBI*, rapid shallow breathing index, *Ppeak* peak airway pressure, *MAP* mean airway pressure, *Pplat*, plateau pressure, *∆P* driving pressure, *Crs* respiratory system compliance, *PMI* pressure-muscle-index

### Respiratory effort and mechanics

**Fig. 1:**
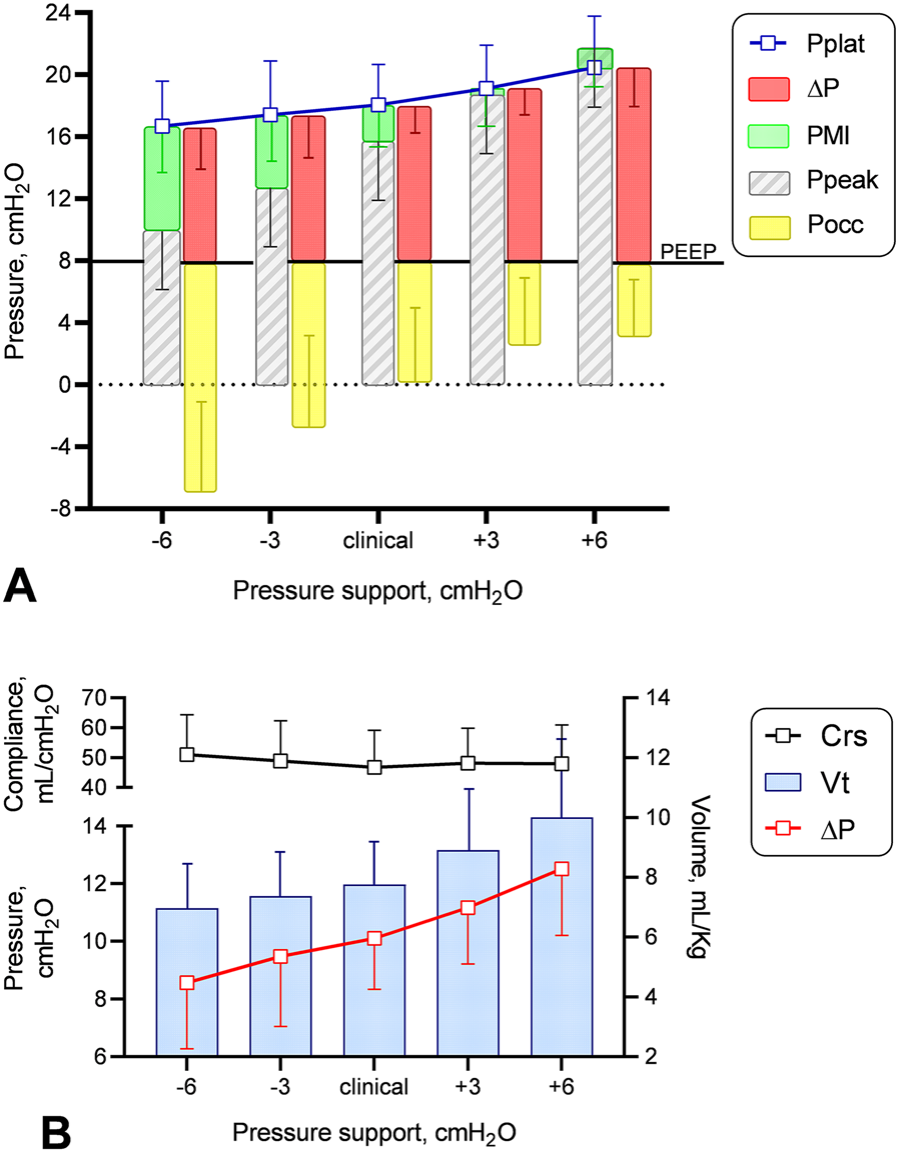
Data obtained with inspiratory and expiratory hold maneuvers in the five PS steps in the study population (*n* = 18). At each PS step, the respiratory system distending pressure, i.e. plateau pressure (Pplat), equals the sum of peak airway pressure plus pressure-muscle-index (PMI). Positive end-expiratory pressure (PEEP) was left unchanged during the study protocol (Fig. 1A). Since respiratory system static compliance (Crs) did not significantly vary across the study steps (*P* = 0.119), tidal volume (Vt) varied proportionally to the applied driving pressure (∆P) and reached values above 8 mL/Kg when PS was raised above the clinically set (Fig. 1B). Data are presented as mean ± SD. Ppeak, peak airway pressure

**Fig. 2:**
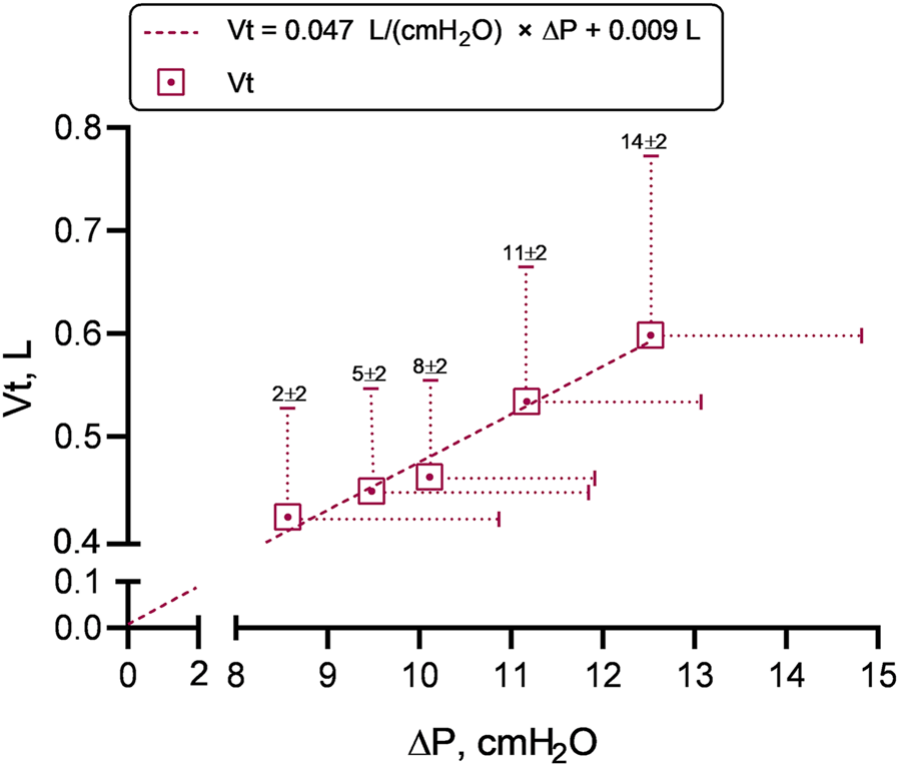
Relationships between tidal volume (Vt) and driving pressure (∆P), i.e. respiratory system compliance (Crs), measured in the five pressure support (PS) steps. Respective PS values in cmH_2_O are reported above each point as mean ± SD rounded to the unit. The regression line of Crs measurements is represented in dotted. Crs did not change among steps (*P* = 0.119) irrespectively of the level of assistance applied in the PS range tested. Data are presented as mean ± SD

Figure 1 shows a summary of the main data obtained from the inspiratory and expiratory holds performed at each PS level. Pplat equals the sum of Ppeak (displayed on the ventilator screen, essentially PEEP + PS) and PMI (unveiled by the inspiratory hold), while ∆P is the difference between Pplat and PEEP (Fig. 1A). As the PS level was increased, PMI progressively decreased (from 6.4 ± 3.2 to −1.2 ± 2.3 cmH_2_O, *P* < 0.001): hence Pplat (from 16.7 ± 2.9 to 20.5 ± 3.3 cmH_2_O, *P* < 0.001) and ∆P (from 8.6 ± 2.3 to 12.5 ± 2.3 cmH_2_O, *P* < 0.001) showed a much smaller variation than Ppeak (from 9.7 ± 3.8 to 21.7 ± 3.8 cmH_2_O, *P* < 0.001). Pocc (from −14.8 ± 6.4 to −4.8 ± 3.5 cmH_2_O, *P* < 0.001) showed a moderate negative correlation with PMI (r = −0.68; CI 95% −0.79 to −0.54; *P* < 0.001), as P0.1 did (*r* = 0.64; CI 95% −0.76 to −0.49; *P* > 0.001)** (**Additional file 1: Figure A4). Since, upon achievement of a relaxed condition, ∆P is the pressure distending the respiratory system, it increased progressively with Vt (from 6.9 ± 1.5 to 10.0 ± 2.6 mL/Kg_PBW_, *P* < 0.001) (Fig. 1B) and Crs did not significantly change (P = 0.119) irrespective of the level of assistance applied in the PS range tested. As a confirm, the intercept of the regression line of the five Crs measurements (*Vt* = *0.047 L/cmH*_*2*_*O x ∆P* + *0.009 L*) was close to zero (Fig. 2).

### Surface electromyography and respiratory drive

Both EADi,surf (from 191 ± 78% to 70 ± 27% of the electrical activity at clinical PS, *P* < 0.001) and intercost,surf (from 150 ± 51% to 74 ± 38% of the electrical activity at clinical PS, *P* < 0.001) signals significantly decreased with increasing PS. Similarly, P0.1 (from 2.5 ± 1.2 to 0.8 ± 0.8, *P* < 0.001) showed an increased respiratory drive when PS was decreased. As shown in the online Additional Content (Additional file 1: Figure A5), EADi,surf correlated with both PMI (*r* = 0.61 95% CI 0.48–0.74; *P* < 0.001; *n* = 90) and P0.1 (*r* = 0.47; 95% CI 0.28–0.66; *P* < 0.001; *n* = 90).

### Individual responses

**Fig. 3:**
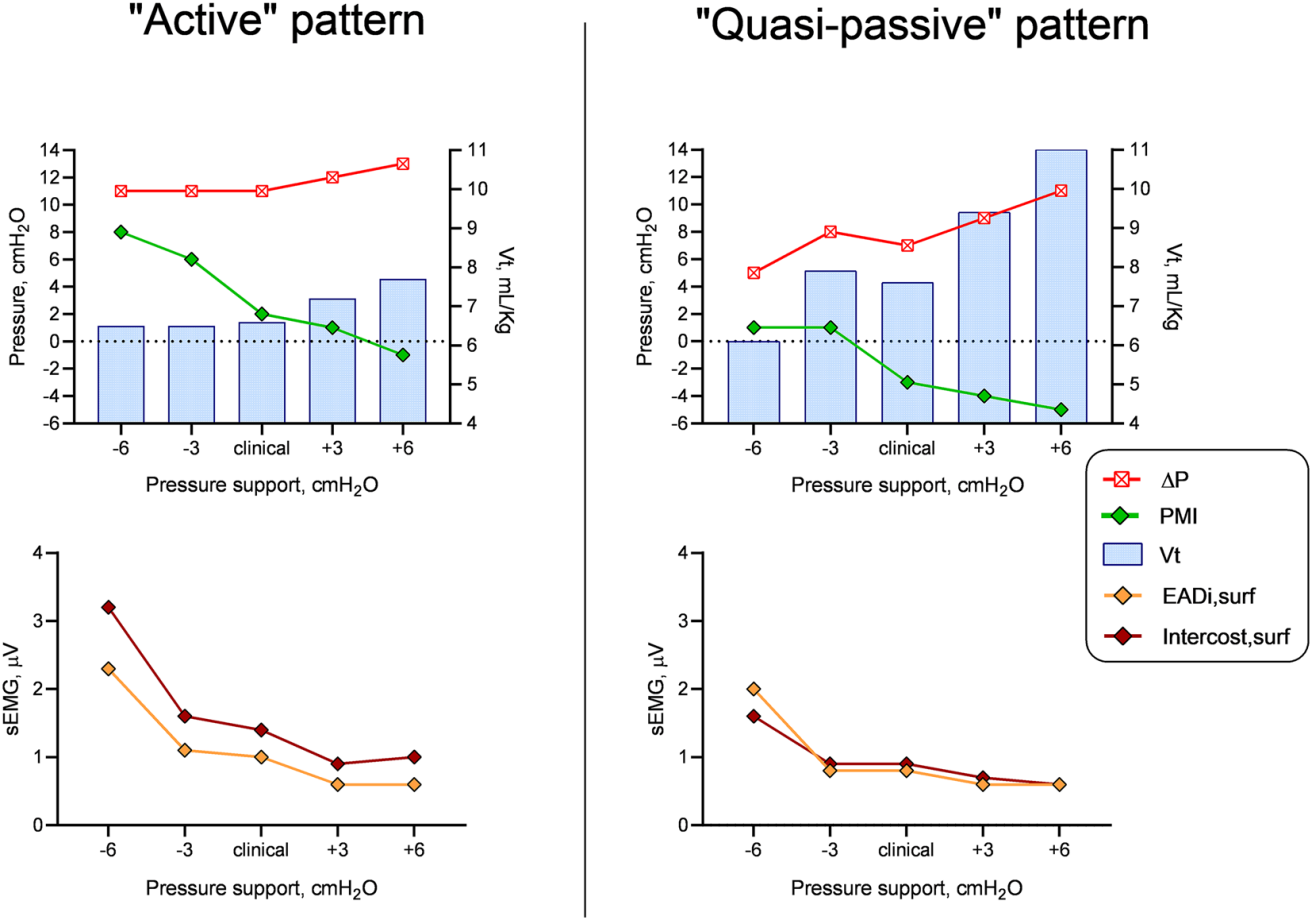
Patterns of response changes in pressure support (PS) level in two representative patients. Active patients react to a consistent decrease in pressure support by developing pressure by their respiratory muscles (pressure-muscle-index, PMI), hence keeping plateau pressure (Pplat) and tidal volume (Vt) relatively constant (upper left panel). On the opposite, a “quasi-passive patient” is almost passively inflated by the ventilator after opening the trigger (upper right panel). When PS is decreased, a fall in Pplat and Vt is observed. Surface electromyography (sEMG) data (lower panels) are consistent with this interpretation. ∆P, driving pressure; EADi,surf, surface electromyography of the diaphragm; Intercost,surf, surface electromyography of the intercostal muscles

**Fig. 4:**
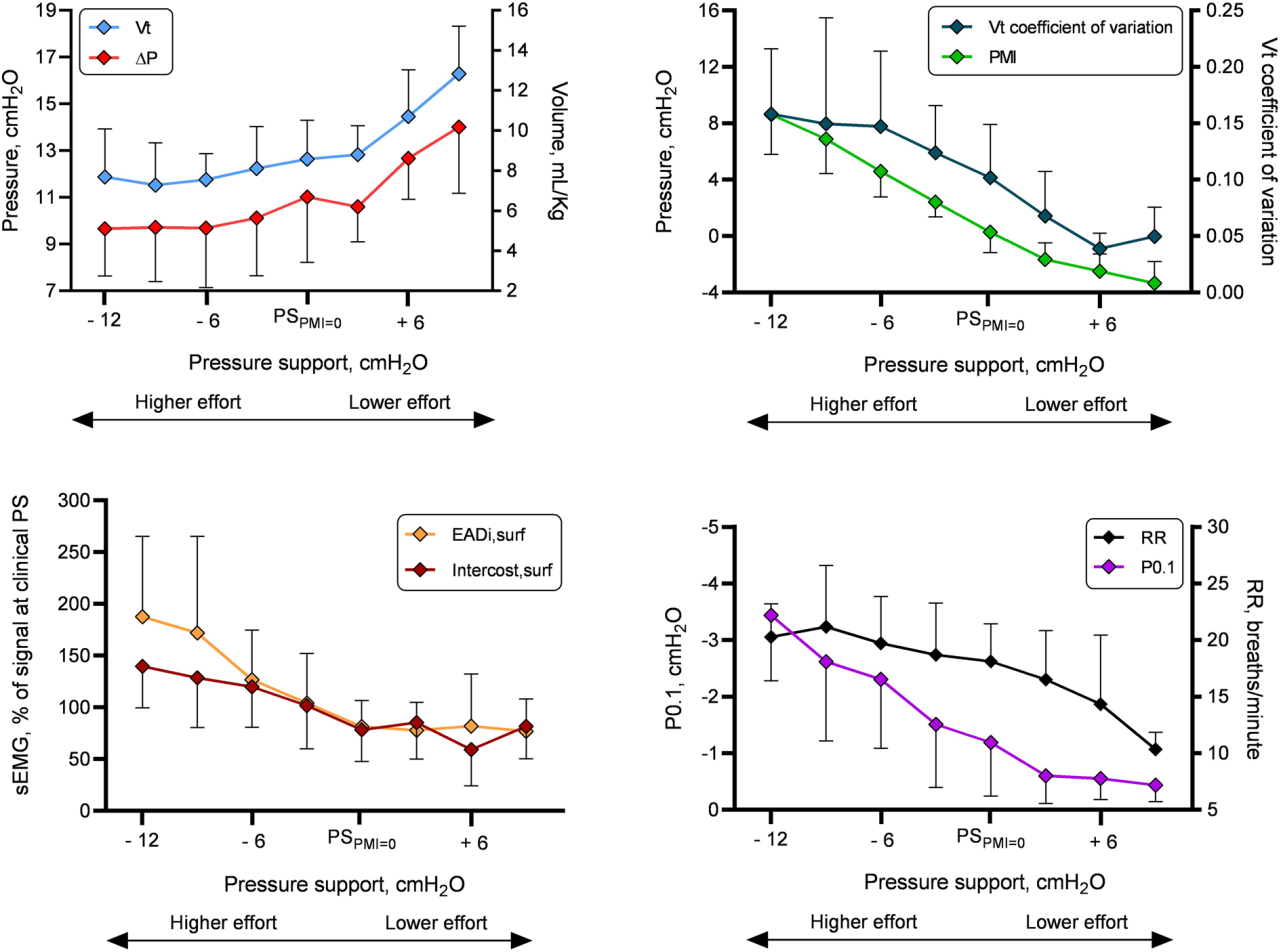
Driving pressure (∆P), tidal volume (Vt) coefficient of variation, respiratory drive and electrical activity of diaphragm at the costal margin (EADi,surf) and intercostal muscles (Intercost,surf) results after a conceptual reorganization on a common “pressure-muscle-index (PMI) axis”. To consider the different individual responses to variation of assistance, data were reorganized so that the five PS levels studied in each patient were shifted, taking as a common reference the PS level at which PMI was the closest possible to disappear (PS_PMI=0_) therefore obtaining a total of 8 PS “bins”, each containing a certain number of the 18 patients studied. Data are shown as mean ± SD. RR, respiratory rate

At an individual level, patients showed different responses to the variation of PS. Some reacted to a decrease in PS by increasing PMI and EADi,surf signal, hence keeping Vt and Pplat almost constant. Vice versa, other patients (which we classified as “quasi-passive” and likely overassisted) were unable to increase PMI due to a poor activation of the inspiratory muscle, remaining almost passively inflated after triggering the ventilator; therefore, the decrease of PS level was associated with a fall in Vt and Pplat. Fig. 3 shows data from two representative patients. To consider the different individual responses to variation of assistance, we reorganized data on a common “PMI axis”, so that the five PS levels studied in each patient were shifted, taking as a common reference the PS level at which the PMI was closest possible to zero (PS_PMI=0_). We therefore obtained a total of 8 PS “bins”, each containing a certain number of the 18 patients studied, so that subsequent bins represent PS variations of 3 cmH_2_O (Additional file 1**:** Figure A6). Additional file 1: Figure A7 shows for each patient the different PS levels through the study steps (either their absolute value and as compared to PS_PMI=0_) with the corresponding PMI value. As shown in Fig. 4, when the level of ventilatory assistance was high enough to zero PMI, both EADi,surf and intercost,surf flattened while Vt and ∆P increased steadily, like in a pressure-controlled mode. Contrarily, when PS was lower than PS_PMI=0_, signal from diaphragm and intercostal muscles electromyography raised while ∆P and Vt remained nearly constant and did not mirror the decrease in PS, as if patients were trying to keep a desired target tidal volume. As a proof of concept, in both cases RR and P0.1 matched the behavior of PMI and sEMG signal, while Vt variability was proportional to inspiratory effort.

### Stratification by higher versus lower Crs

To interpret our findings based on common respiratory patterns of non-obstructive respiratory failure (i.e., restrictive pattern or with a higher percentage of aerated lung), as an exploratory approach, patients were divided in two subgroups based on a Crs lower or higher (respectively 37 ± 7 mL/cmH_2_O and 57 ± 7 mL/cmH_2_O) than the population median value. Respiratory mechanics parameters for each subgroup are presented in the Supplemental Digital Content (Additional file 1: Table A2). Additional file 1: Figure A8 shows Vt and ∆P at the different PS levels in the two subgroups. As for the whole population, Crs did not significantly change within higher (*P* = 0.195) or lower (*P* = 0.281) Crs subgroups through the five PS steps. Patients with higher Crs showed a wider range of ∆P (from 7.6 ± 2.0 to 12.7 ± 2.5 cmH_2_O, *P* < 0.001) and Vt (from 7.2 ± 1.3 to 11.8 ± 1.8 mL/Kg, *P* < 0.001) than the lower Crs group (*P* < 0.05), even if the highest ∆P-values were not significantly different between the subgroups (12.3 ± 2.3 cmH_2_O for lower and 12.7 ± 2.5 cmH_2_O for higher Crs group; P = 0.802).

## Discussion

The main findings of this study can be summarized as follows: while previous studies reported that an increase in the level of assistance leads to a decrease of patient’s effort [30, 31], we show through a systematic assessment with inspiratory holds that in these circumstances effort decreases until a level at which the patient becomes “quasi-passive” (as indicated by a PMI of 0 cmH_2_O). At this point, for a given PS, the patient’s Crs becomes the only determinant of Vt, similarly (except from trigger) to what happens to paralyzed patients in pressure-controlled ventilation.

Cammarota and colleagues recently described that, when ventilating in PSV at higher levels of assistance, Vt tends to exceed the threshold of 8 mL/Kg_PBW_ [32]. We show that this behavior may be more relevant in patients with higher Crs, where PMI could represent a useful tool in setting the level of assistance avoiding over-assistance and excessive, potentially harmful, ventilation. When set PS is high enough to exceed the PS level corresponding to a PMI of 0 cmH2O (PS_PMI=0_), the excess of volume over the patient’s desired target can be computed as the product of Crs and the difference between set PS and PS_PMI=0_, up to the point where the lungs become overdistended and the increase in Vt potentially leads to a non-protective ventilation. Further studies might clarify which exact thresholds should be applied to consider assisted spontaneous ventilation as being protective.

Instead, when the level of assistance is decreased, an “active” patient with preserved muscular strength and respiratory drive is expected to react by increasing the muscular pressure (i.e., PMI) and maintain the desired Vt. Therefore, it is often not possible to lower ∆P below the ratio between that Vt and Crs by simply decreasing PS on the ventilator. Conversely, when a patient is not able to generate enough muscular pressure to cope with reduction in assistance, as in case of muscle weakness, Vt would progressively decrease due to the lack of PMI increase.

Our group and others have already described the presence of a moderate-to-strong correlation between the electrical activity of the diaphragm measured with sEMG and that measured with a nasogastric catheter [22] or Pes-derived PTP [33] at different levels of ventilatory assistance. In addition to what already described, our study shows that EADi,surf correlates with both PMI and P0.1 (i.e., a well-established index of respiratory drive); thus it could be considered as a global index of inspiratory effort. When over-assisting patients with excessive PS, sEMG signal (as PMI) is abolished, suggesting that the threshold of patient’s desired Vt has been overcome. In contrast sEMG signal raises proportionally with respiratory effort and drive when patients are well- or under assisted.

Moreover, we corroborate the reliability of the measurement of Crs through an end inspiratory hold during PSV, showing that ∆P is only a function of Vt irrespectively from the level of assistance applied. Our group and others have already shown that it is possible to measure ∆P during assisted ventilation [34–36], and that this value correlates with severity of lung injury and outcome [20]. The value of Pplat during assisted ventilation is often higher than Ppeak, since it also includes the elastic pressure deriving from relaxation of inspiratory muscles (PMI). Readability criteria have been recently described [29] but measurement reliability of PPlat might be hampered by a hidden muscle effort present despite a flat plateau. Our finding that Crs is constant over a wide range of ventilatory assistance supports the reliability of the measurement of Pplat during patient triggered breaths, in that, while Ppeak varies widely, Pplat (and hence ∆P) remains fairly constant, until Vt does not change.

This study has several limitations which need to be considered [1]. The number of patients is relatively small and enrolled in a single center, but such setting allowed us to perform a rather complex collection of physiological data in a very controlled and standardized way. [2] We applied each level of PS for a relatively short timeframe, i.e., 10 min. Overall, this allowed the experiment to last one hour, avoiding changes in the patient’s condition (i.e., sedation, body temperature, secretions), that would have confounded the comparison between the different phases. However, [3] we cannot exclude the presence of confounding influences of the typical cross-over design because of the completely randomized pattern of PS, such as maturation or carry-over effects. [4] Many ICU ventilators cannot deliver a PS lower than 2 cmH_2_O even if it is set on the screen by the clinician. For this reason, the difference in inspiratory support between step -3 and -6 may be less than expected. [5] The studied population was relatively mild, as indicated by the oxygenation value and the lack of respiratory acidosis, with over assistance likely present in some patients. While these conditions are representative of several patients after the early, acute phase, this limits our findings which might not be generalizable. [6] It is also possible that, these circumstances facilitated the relaxation of patients at end-inspiration, so that it was possible to obtain a readable hold at all the levels of assistance. Again, this might not be applicable to all patients undergoing Pressure Support Ventilation, especially to those with a higher respiratory drive. Moreover, since only steps of 3 cmH_2_O of PS were tested, the exact threshold to define overassistance (i.e., PS_PMI=0_) could sometimes not be known and was hence approximated to the step whose PMI was closest to zero. [7] We did not measure the reference standard for breathing effort, i.e., Pes. This can be also seen as a strength of the study, as we have been able to fully characterize patient’s response and respiratory mechanics from airway pressure signal and sEMG, which is known to correlate with diaphragmatic activity [22]. Finally, [8] we did not assess the presence of intrinsic PEEP, which slightly varies on a breath-to-breath basis in spontaneous breathing, potentially leading to an overestimation of ∆P, and is measurable by means of esophageal manometry. Furthermore, as the estimation of inspiratory effort with PMI considers only the elastic pressure developed by the patient’s inspiratory muscles, it may not be suitable for patients with increased airway resistance (condition often concomitant to intrinsic PEEP). For these reasons, we excluded from the study patients whose etiology for being mechanically ventilated was due to airway resistances abrupt increase (see Methods).

In conclusion we describe the response to a change in ventilatory assistance in which PMI varies to keep Vt and ∆P close to a specific patient’s desired value. If the assistance increases to a level that abolishes PMI, patients lose control over Vt like in a pressure-control mode. PMI might be a useful tool in evaluating the presence of overassistance. Moreover, the measurement of Crs remains constant independently from the level of assistance and (hence) patient’s effort.

### Supplementary Information

**Additional file 1.** Methods to sEMG positioning and data acquisition and processing, additional figures and tables.

## Data Availability

The datasets used and/or analyzed during the current study are available from the corresponding author on reasonable request.

## Abbreviations

ARDS: Acute respiratory distress syndrome
Crs: Respiratory system static compliance
∆P: Driving pressure
EADi,surf: Surface electromyography of the diaphragm
FiO_2_: Inspiratory oxygen fraction
Intercost,surf: Surface electromyography of the intercostal muscles
MV: Minute ventilation
PaO_2_: Oxygen arterial partial pressure
PaCO_2_: Carbon dioxide arterial partial pressure
PEEP: Positive end-expiratory pressure
Ppeak: Peak airway pressure
Pplat: Plateau pressure
PMI: Pressure-muscle-index
PS_PMI=0_: Pressure support level at which pressure-muscle-index is zero
PSV: Pressure support ventilation
RR: Respiratory rate
sEMG: Surface electromyography
SOFA: Sequential organ failure assessment
Vt: Tidal volume
