# Supplementary material for: Individual response in patient’s effort and driving pressure to variations in assistance during pressure support ventilation
Source: Ann Intensive Care. 2023 Dec 20;13:132. doi: 10.1186/s13613-023-01231-9 (PMC10733248; doi:10.1186/s13613-023-01231-9)
Supplement: Supplementary file 1 — Additional file 1. Methods to sEMG positioning and data acquisition and processing, additional figures and tables. [file 13613_2023_1231_MOESM1_ESM.docx]

**Individual response in patient’s effort and driving pressure to variations in ventilatory assistance. Additional file 1.**

**Materials and methods**

**Surface electromyography**

The surface electromyography signals were collected through two pairs of ECG surface electrodes (Kendall 530 Foam Electrodes, Covidien, Dublin, Ireland). **Figure A2** in the online supplements shows the exact electrodes position (1): one pair was placed bilaterally at the crossing point between the subcostal margin (below the 12th rib bilaterally) and the midclavicular line to sense the diaphragm electrical activity (EADi,surf); the other bilaterally at the level of the second intercostal space on the parasternal line to sense the intercostal muscles electrical activity (Intercost,surf). The ground electrode was placed at the xiphoid. These electrodes were connected to a dedicated device (sEMG Recorder, Drägerwerk AG & Co., Lubeck, Germany), that records also outputs from the ventilator (airway pressure and flow traces). Data recorded during the study were stored for off-line analysis. Once removed electromyography artifacts with a dedicated software by Draeger, traces were analyzed using LabChart 7 Pro (ADInstruments, Sidney, Australia). For each patient 75 respiratory cycles were selected (15 for each PS step), excluding segments affected by artifacts (e.g. coughing) or electromyography signal instability. For each selected respiratory cycle, we calculated the difference between maximum and minimum voltage in the inspiratory phase for both diaphragm and intercostals signal. For each patient, at each PS level, the mean differences between maximum and minimum values of electrical activity of the diaphragm at the costal margin and the intercostal muscles were then expressed as percentage of the signal measured at clinical PS (%clin).

**Additional Tables and Figures**

| Table A1. Demographics and baseline characteristics. | |
| --- | --- |
| Characteristic | Patients (n=18) |
| Age, years | 60 ± 14 |
| Female sex, n° (%) | 11 (61) |
| Weight, Kg | 78 ± 20 |
| Height, cm | 165 ± 6 |
| BMI, Kg/m^2^ | 28 ± 8 |
| Comorbidities |  |
| Hypertension, n° (%) | 6 (33) |
| CAD, n° (%) | 3 (16) |
| Chronic Kidney Disease, n° (%) | 3 (16) |
| Immunoincompetence, n° (%) | 5 (27) |
| Diabetes, n° (%) | 2 (12) |
| Obesity, n° (%) | 5 (27) |
| Reason for ICU admission |  |
| AHRF, n° (%) | 8 (44) |
| ARDS, n° (%) | 3 (16) |
| Septic shock, n° (%) | 3 (16) |
| Trauma, n° (%) | 2 (12) |
| Surgery, n° (%) | 2 (12) |
| Baseline characteristics |  |
| Days from intubation, n° | 6 [4;12] |
| SOFA score on study day | 6 [3;8] |
| Body temperature, °C | 37.4 ± 0.6 |
| RASS | -1 [-2;0] |
| FiO_2,_ % | 38 ± 8 |
| PaO_2_ | 98 ± 19 |
| PaO_2_/FiO_2_ | 264 ± 84 |
| Arterial pH | 7.46 ± 0.04 |
| PaCO_2_, mmHg | 43 ± 5 |
| BE, mmol/L | 5.5 ± 3.7 |
| Serum lactate level, mmol/L | 1.33 ± 0.48 |

**Table A1**. Data are presented as mean ± SD or median [IQR] as appropriate. BMI, body mass index; CAD, coronary artery disease; Immunoincompetence, presence of either drugs or neoplasms able to suppress the immune system or a congenital immune disease; ICU, intensive care unit; obesity was defined as BMI>30 Kg/m^2;^ AHRF, acute hypoxic respiratory failure; ARDS, acute respiratory distress syndrome; SOFA score, sequential organ failure assessment score; RASS, Richmond agitation-sedation scale; FiO_2_, oxygen inspiratory fraction; PaO2~~,~~ oxygen partial pressure; PaCO2, carbon dioxide arterial partial pressure; BE, base excess.

| Table A2. Secondary analysis: respiratory mechanics in low and high static respiratory system compliance subgroups. | | | | | | |
| --- | --- | --- | --- | --- | --- | --- |
| Lower respiratory system compliance | | | | | | |
|  | -6 cmH_2_O | -3 cmH_2_O | Clinical PS | +3 cmH_2_O | +6 cmH_2_O | P value |
| PS, cmH_2_O | 0.9 ± 1.5 | 3.9 ± 1.5 | 6.7 ± 1.5 | 9.9 ± 1.5 | 12.9 ± 1.5 | <0.001 |
| PEEP, cmH_2_O | 7.1 ± 1.9 | 7.1 ± 1.9 | 7.1 ± 1.9 | 7.1 ± 1.9 | 7.1 ± 1.9 | N/A |
| Vt, L | 0.39± 0.11 | 0.40± 0.11 | 0.39± 0.06 | 0.45± 0.11 | 0.46± 0.11 | 0.079 |
| Vt, mL/Kg | 7.2 ± 2.2 | 7.4 ± 2.3 | 7.2 ± 1.3 | 8.1 ± 2.0 | 8.4 ± 2.2 | 0.082 |
| MV, L/min | 8.5 ± 1.5 | 8.4 ± 1.5 | 8.3 ± 0.9 | 8.2 ± 1.0 | 9.0 ± 1.7 | 0.648 |
| RR, breaths/min | 23 ± 4 | 21 ± 5 | 21 ± 5 | 18 ± 3 | 17 ± 4 | 0.026 |
| RSBI, breaths/min/L | 55 ± 20 | 53 ± 20 | 57 ± 27 | 41 ± 13 | 38 ± 16 | <0.001 |
| Ppeak, cmH_2_O | 8.6 ± 2.7 | 11.2 ± 2.7 | 14.2 ± 2.7 | 17.2 ± 2.7 | 20.3 ± 2.7 | <0.001 |
| Pplat, cmH_2_O | 17.2 ± 2.9 | 18.0 ± 3.4 | 18.1 ± 2.0 | 18.5 ± 1.9 | 19.7 ± 2.7 | 0.021 |
| ∆P, cmH_2_O | 9.6 ± 2.2 | 10.7 ± 2.4 | 10.8 ± 1.2 | 11.2 ± 1.3 | 12.3 ± 2.3 | 0.011 |
| Crs, mL/cmH_2_O | 42 ± 11 | 38 ± 6 | 37 ± 7 | 40 ± 10 | 38 ± 8 | 0.195 |
| PMI, cmH_2_O | 8.4 ± 2.9 | 6.9 ± 2.7 | 3.8 ± 2.4 | 1.2 ± 2.0 | -0.5 ± 2.7 | <0.001 |
| P0.1, cmH_2_O | 3.1 ± 1.2 | 2.4 ± 1.5 | 1.8 ± 1.3 | 1.3 ± 1.2 | 1.0 ± 1.0 | <0.001 |
| EADi,surf, %clin | 178 ± 80 | 139 ± 81 | 100 ± 0 | 78 ± 23 | 76 ± 29 | 0.011 |
| Intercost,surf, %clin | 135 ± 42 | 111 ± 22 | 100 ± 0 | 72 ± 27 | 73 ± 38 | 0.003 |
| Higher respiratory system compliance | | | | | | |
|  | -6 cmH_2_O | -3 cmH_2_O | Clinical PS | +3 cmH_2_O | +6 cmH_2_O | P value |
| PS, cmH_2_O | 2.7 ± 2.2 | 5.6 ± 2.2 | 8.6 ± 2.2 | 11.7 ± 2.2 | 14.7 ± 2.2 | <0.001 |
| PEEP, cmH_2_O | 8.6 ± 3.0 | 8.6 ± 3.0 | 8.6 ± 3.0 | 8.6 ± 3.0 | 8.6 ± 3.0 | N/A |
| Vt, L | 0.45 ± 0.09 | 0.49 ± 0.08 | 0.53 ± 0.08 | 0.62 ± 0.10 | 0.73 ± 0.09 | <0.001 |
| Vt, mL/Kg | 7.2 ± 1.3 | 7.9 ± 1.2 | 8.5 ± 1.2 | 10.0 ± 1.7 | 11.8 ± 1.8 | <0.001 |
| MV, L/min | 8.6 ± 2.3 | 8.6 ± 1.5 | 8.9 ± 1.8 | 8.6 ± 1.7 | 8.8 ± 1.5 | 0.962 |
| RR, breaths/min | 21 ± 4 | 18 ± 3 | 17 ± 3 | 15 ± 3 | 13 ± 3 | <0.001 |
| RSBI, breaths/min/L | 49 ± 18 | 37 ± 12 | 31 ± 9 | 26 ± 9 | 18 ± 6 | <0.001 |
| Ppeak, cmH_2_O | 11.2 ± 4.2 | 14.2 ± 4.2 | 17.2 ± 4.2 | 20.2 ± 4.2 | 23.2 ± 4.2 | <0.001 |
| Pplat, cmH_2_O | 16.1 ± 2.9 | 16.8 ± 3.6 | 18.0 ± 3.3 | 19.7 ± 3.5 | 21.3 ± 3.7 | <0.001 |
| ∆P, cmH_2_O | 7.6 ± 2.0 | 8.3 ± 1.8 | 9.4 ± 2.0 | 11.1 ± 2.5 | 12.7 ± 2.5 | <0.001 |
| Crs, mL/cmH_2_O | 60 ± 7 | 60 ± 8 | 57 ± 7 | 56 ± 7 | 58 ± 7 | 0.281 |
| PMI, cmH_2_O | 4.4 ± 2.2 | 2.6 ± 2.1 | 0.8 ± 2.1 | -0.5 ± 2.2 | -1.9 ± 1.5 | <0.001 |
| P0.1, cmH_2_O | 2.3 ± 1.4 | 2.0 ± 1.4 | 1.3 ± 0.9 | 1.0 ± 0.9 | 1.0 ± 1.1 | 0.017 |
| EADi,surf, %clin | 204 ± 78 | 129 ± 34 | 100 ± 0 | 76 ± 39 | 64 ± 26 | 0.001 |
| Intercost,surf, %clin | 166 ± 56 | 115 ± 35 | 100 ± 0 | 67 ± 27 | 75 ± 40 | 0.003 |

**Table.A2** Data are presented as mean ± SD. P values shown correspond to one-way ANOVA for repeated measurements significance level. Surface electromyography of diaphragm (EADi,surf) and intercostal muscles (Intercost,surf) is expressed as percentage of the signal at clinical PS (%clin). PS, pressure support; PEEP, positive end expiratory pressure; Vt, tidal volume; MV, minute ventilation; RR, respiratory rate; RSBI, rapid shallow breathing index; Ppeak, peak airway pressure; Pplat, plateau pressure; ∆P, driving pressure; Crs, respiratory system compliance; PMI, pressure-muscle-index.

| Table A3. Surface electromyography of the diaphragm in the five PS steps tested. | | | | | | |
| --- | --- | --- | --- | --- | --- | --- |
|  | -6 cmH_2_O | -3 cmH_2_O | Clinical PS | +3 cmH_2_O | +6 cmH_2_O | P value |
| EADi,surf, %clin | 191 ± 78 | 134 ± 61 | 100 ± 0 | 77 ± 31 | 70 ± 27 | <0.001 |
| EADi,surf, μV | 3.9 ± 2.6 | 2.9 ± 2.5 | 2.3 ± 2.0 | 1.5 ± 1.1 | 1.4 ± 1.2 | <0.001 |
| Signal-to-noise ratio | 3.4 ± 1.8 | 3.1 ± 1.9 | 2.6 ± 1.6 | 2.1 ± 1.1 | 2.0 ± 1.2 | <0.001 |

**Table A3** Diaphragm surface electromyography (EADi,surf) in the five PS steps tested. Data are presented as % of signal at clinical PS (%clin) as well as raw data (μV). Signal-to-noise ratio expresses the relationship between peak and baseline of electrical activity in μV. Data are expressed as mean ± SD. P-values shown correspond to one-way ANOVA for repeated measurements significance level.


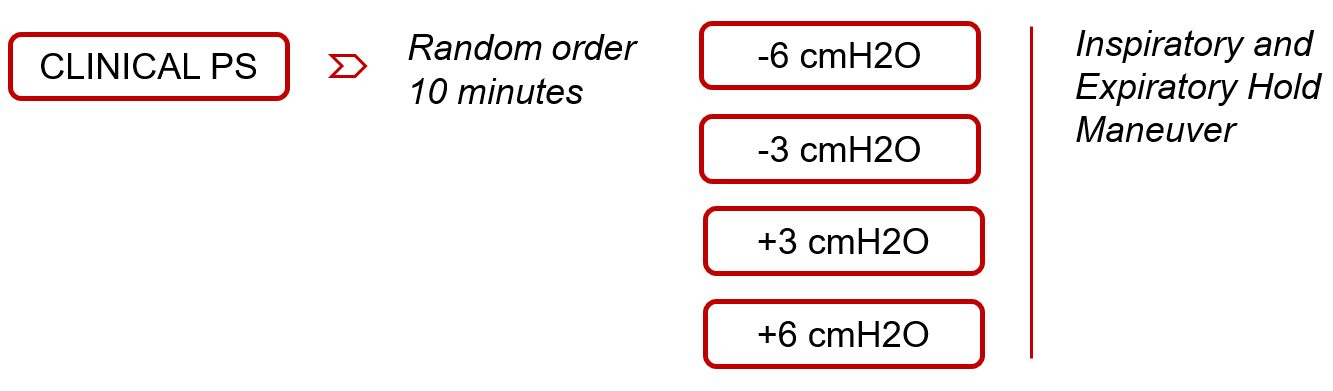


**Figure A1** Study design. Five PS steps were delivered to patients in random order, starting from the clinical PS. In each PS step inspiratory and expiratory holds were performed to measure inspiratory effort and respiratory mechanics.

**Figure A2** Ventilator and surface electromyography waveforms in a representative patient at clinical pressure support (PS) compared to step -6 and +6 cmH_2_O. A good quality end inspiratory hold allows to obtain the total elastic pressure applied to the respiratory system (i.e. plateau pressure, Pplat) and the dynamic elastic pressure generated above the end expiratory lung volume (i.e. driving pressure, ∆P). PS, pressure support; Ppeak, peak airway pressure; Pplat, plateau pressure; PMI, pressure-muscle-index; Paw, airway pressure; EADi,surf, surface electromyography of the diaphragm.

**
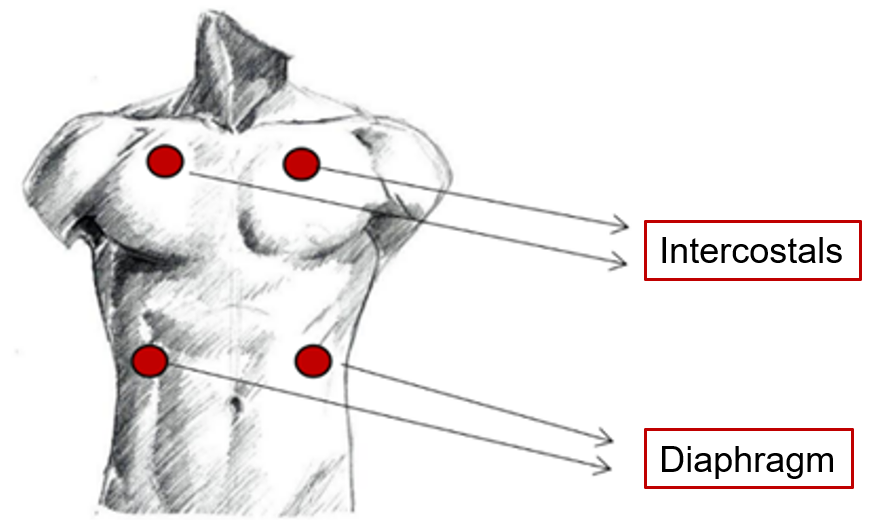
**

**Figure A3.** Surface electromyography electrodes configuration for diaphragm and intercostal muscles. The ground electrode was placed at the xiphoid.

**Figure A4** Pressure-muscle-index (PMI) correlation with Pocc and P0.1 in the study population. Correlation is expressed as Pearson index (r).

**Figure A5** Surface electromyography of the diaphragm (EADi, surf) correlates with pressure-muscle-index (PMI), P0.1 and Pocc. Patients at different PS levels are represented via a spaghetti plot with proper regression lines (grey lines) plus overall population regression lines with 95% confidence interval (green for pressure-muscle-index; gold for P0.1; purple for Pocc) and Spearman correlation index (r) with its significance level (P).


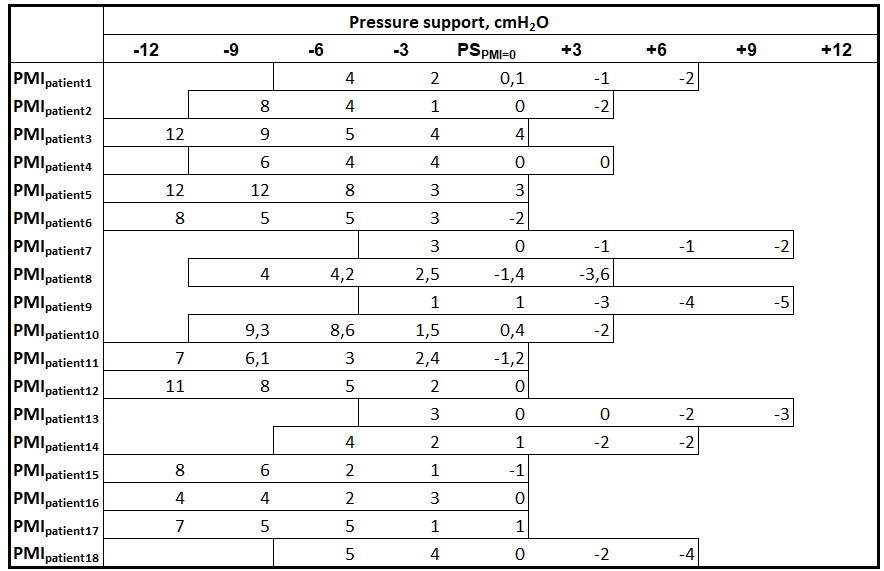


**Figure A6** To consider the several different individual responses to a variation of the level of ventilatory assistance, we reorganized data on a common “pressure-muscle-index (PMI) axis”, so that the five PS levels studied in all patients were shifted, taking as a common reference the level where PMI was closest possible to zero (PS_PMI=0_), leading to a total of eight PS steps “bins”. Subsequent bins represent PS variations of 3 cmH_2_O.

Figure A7. Spaghetti plot illustrating for each patient the different Pressure Support (PS) levels through the study steps with the corresponding pressure-muscle-index (PMI) value. PS levels are displayed as either absolute value (left panel) and as compared to the PS level at which PMI was closest to zero (right panel) - PSMPMI=0.

**
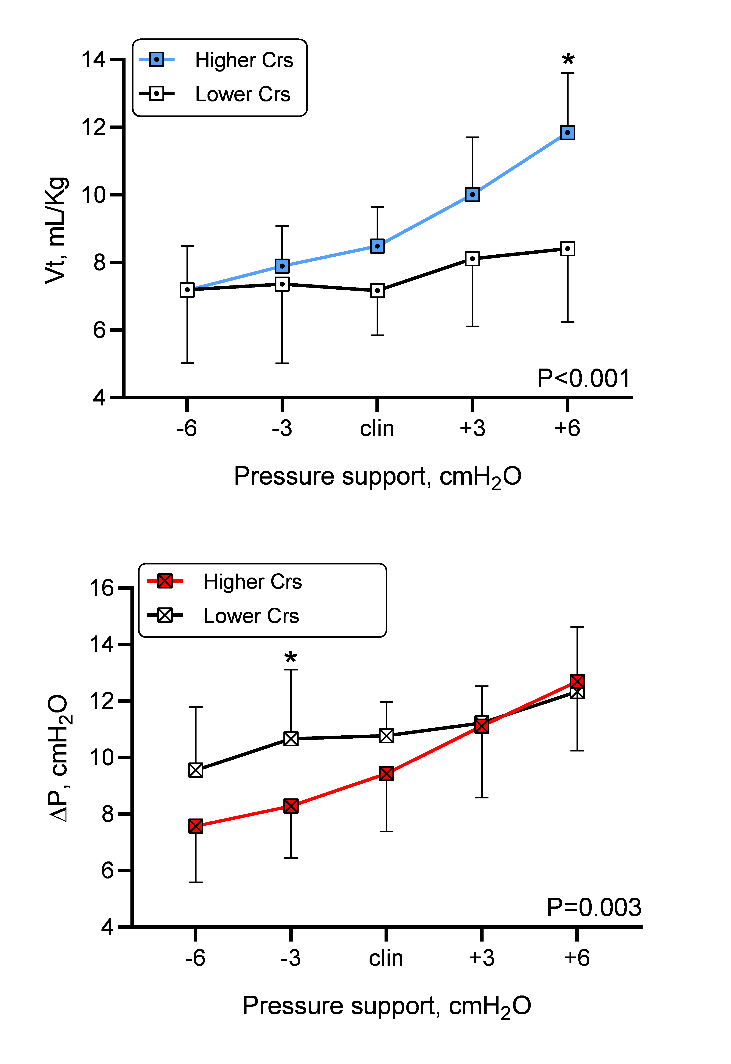
**

**Figure A8.** Driving pressure (∆P) and tidal volume (Vt) in the five pressure support (PS) steps in low and high respiratory system compliance (Crs) subgroups. Patients with higher Crs showed a wider range of ∆P and Vt. Vt exceeded 8 mL/Kg when patients with higher Crs were ventilated with a PS equal or greater than the clinically set. Data are presented as mean ± SD. P, mixed ANOVA significance level for interaction; *, two-stage linear step-up procedure of Benjamini, Krieger and Yekutieli significance level <0.05 for high versus low Crs.

**References:**

1. Pozzi M, Rezoagli E, Bronco A, Rabboni F, Grasselli G, Foti G, et al. Accessory and Expiratory Muscles Activation During Spontaneous Breathing Trial: A Physiological Study by Surface Electromyography. Front Med. 2022;9(March):1–11.
